# Extrinsic Contribution and Instability Properties in Lead-Based and Lead-Free Piezoceramics

**DOI:** 10.3390/ma8115426

**Published:** 2015-11-19

**Authors:** José Eduardo García

**Affiliations:** Department of Physics, Universitat Politècnica de Catalunya—BarcelonaTech., 08034 Barcelona, Spain; jose.eduardo.garcia@upc.edu; Tel.: +34-934-016-086; Fax: +34-934-016-090

**Keywords:** piezoelectric materials, nonlinear properties, extrinsic contribution, dielectric properties, piezoelectric properties

## Abstract

Piezoceramic materials generally exhibit a notable instability of their functional properties when they work under real external conditions. This undesirable effect, known as nonlinear behavior, is mostly associated with the extrinsic contribution to material response. In this article, the role of the ferroelectric domain walls’ motion in the nonlinear response in the most workable lead-based and lead-free piezoceramics is reviewed. Initially, the extrinsic origin of the nonlinear response is discussed in terms of the temperature dependence of material response. The influence of the crystallographic phase and of the phase boundaries on the material response are then reviewed. Subsequently, the impact of the defects created by doping in order to control the extrinsic contribution is discussed as a way of tuning material properties. Finally, some aspects related to the grain-size effect on the nonlinear response of piezoceramics are surveyed.

## 1. Introduction

Piezoelectric ceramics have been widely used in sensor and actuator applications for more than a half century, ranging from sonar to pressure sensors, ultrasonic transducers, fuel injectors, microphones, resonators, filters, and many others [1]. While piezoceramics are parts of a mature technology [2], emerging challenges have stepped up the demand for new high performance piezoelectric materials with specific functionalities. For instance, the miniaturization trend in piezoelectric technologies requires ceramics with submicron or even lower grain size. Furthermore, environmental concerns as well as governmental regulations against hazardous substances have led to the development of new environmentally friendly piezoceramic compositions. More recently, further advances in piezoelectric technology for energy harvesting have demonstrated the need for low cost, high sensitive piezoelectric materials in order to achieve large deformations as a response to low applied voltages.

Lead-based microstructured materials with perovskite structure are the most common active elements in piezoelectric devices because of their excellent properties and relatively low manufacturing costs. In fact, polycrystalline lead zirconate titanate (PZT) has for decades been dominant in the market for piezoelectric materials [3]. The PZT system enjoys high flexibility in terms of compositional modifications, thereby providing a wide spectrum of compositions with improved properties for specific applications. PZT-based materials are classified into two groups, known as “hard” and “soft” according to some particular properties [4]. These terms are commonly used for ranking piezoelectric materials in order to specify their ability to be transferred to applications. Hard materials are characterized by low losses and a high quality factor, but also by moderate values of both dielectric constant and piezoelectric coefficients, while soft materials exhibit high values of dielectric constant and piezoelectric coefficient, but also high losses. Hard materials are typically used in resonant devices where a high mechanical quality factor and a high electromechanical coupling factor are needed, while soft materials are usually used in non-resonant devices where their critical figures of merit are determined by both the piezoelectric strain coefficient and the piezoelectric voltage coefficient.

The dielectric and piezoelectric responses of piezoceramics to either an electrical or a mechanical external stimulus are due to two contributions known as intrinsic and extrinsic. The intrinsic contribution concerns the linear lattice distortion and is associated with the change in the polarization of the unit cell. This change in polarization is related to both the polarization extension and the polarization rotation [5]. On the other hand, the extrinsic contribution is easily defined as all responses that are different from the intrinsic response, which is mainly due to domain wall motion in perovskite polycrystals [6]. Both contributions maximize in the region delimiting different polymorphic phases, such as a morphotropic phase boundary (MPB), thereby improving macroscopic properties of materials [7,8]. The MPB is a compositionally-driven structural change region where the coexistence of two ferroelectric phases enhances the macroscopic properties, which is the reason why high performance piezoelectric materials are MPB systems. This type of phase boundary is also stated as structurally bridging low symmetry regions, which have been described as monoclinic symmetry regions for PZT and other systems [9,10].

Functional properties of piezoceramics are undesirable dependents of external parameters. The dielectric and piezoelectric properties generally exhibit aging, frequency dispersion, and nonlinearity [11]. The term nonlinearity commonly refers to the instability of these properties when a sub-coercive alternating electric field or a dynamic mechanical stress is applied to the material [12,13,14,15]. This nonlinear effect postulates that once the applied field is removed, the material recovers its linear, or low-field, properties. Thus, the electric field is applied in the sub-switching regime, whereas the applied mechanical stress is such that it does not produce depoling. Other phenomena occurring in piezoelectric materials also belong to nonlinear behavior, although they are not addressed in this review. For instance, nonlinearity also refers to: (i) the variation of the elastic properties and the electromechanical coupling factor in resonant conditions, which is essential for establishing the working limits of high-power devices [16,17,18,19]; and, (ii) the decrease in permittivity under a high DC electric field, known as electrical tuning of permittivity, which is the phenomenon on which a large number of microwave devices are based [20,21,22].

Piezoceramic materials show a noticeable nonlinear behavior at a relatively low electric field or dynamical stress [12,14]. Since the dielectric and piezoelectric properties of piezoceramics are strongly determined by the ferroelectric/ferroelastic domain structure and the dynamics of the domain walls [23], the nonlinear response will depend on any microstructural aspect that modifies the domain structure and/or the domain wall motion, such as the crystallographic phase, the grain size, and the compositional modifications by doping. The microstructural dependence of nonlinear response has been widely studied in PZT [24,25,26,27] and other ceramic systems such as barium titanate (BT) [28], lead magnesium niobate—lead titanate (PMN-PT) [29], and potassium sodium niobate (KNN) [30]. This review focuses on the role of the ferroelectric domain wall motion/dynamics in the nonlinear response in the most workable lead-based and lead-free piezoelectric ceramics with a perovskite structure. Moreover, a description is given of how nonlinear measurements may be used as a tool to study the mechanisms contributing to dielectric and piezoelectric properties in these materials.

## 2. Extrinsic Contribution and Nonlinear Response

The intrinsic and extrinsic contributions to material response can be experimentally separated by taking into account their different phenomenological nature. The intrinsic contribution comes from the polarization rotation and polarization extension phenomena [5]. The first is essentially dependent on the crystal structure, whereas the second is primarily important in regions where phase transitions occur. Hence, intrinsic contribution may be considered temperature-independent in temperature ranges that are sufficiently far from phase transitions. In contrast, the extrinsic contribution related to domain wall motion is markedly temperature-dependent. The motion of the domain walls is thermally assisted, and is generally the main contribution to material response at room temperature [6,29,30,31,32]. Consequently, only intrinsic response is expected at very low temperature as a result of the domain wall freezing phenomenon [33].

**Figure 1:**
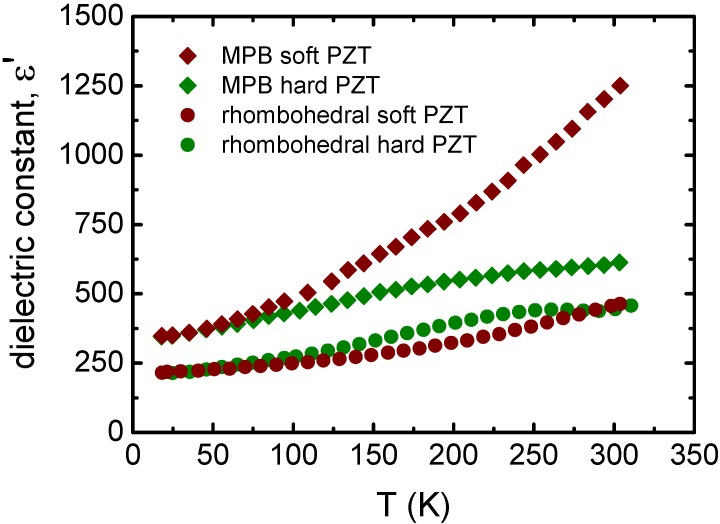
Temperature dependent dielectric constant for Pb(Zr_0.60_Ti_0.40_)O_3_ (rhombohedral PZT) and Pb(Zr_0.53_Ti_0.47_)O_3_ (MPB PZT) doped with Nb^5+^ (soft PZT) or Fe^3+^ (hard PZT) from low temperatures to above room temperature. The data were extracted from the reference [35] (Copyright AIP Publishing LLC., 2007).

Figure 1 shows the temperature dependence of the dielectric constant for Nb- and Fe-doped Pb(Zr_0.60_Ti_0.40_)O_3_ (rhombohedral PZT) and Pb(Zr_0.53_Ti_0.47_)O_3_ (MPB PZT) from low temperatures (~15 K) to above room temperature (~390 K). In all cases, ε′(*T*) increases from an intrinsic low temperature dielectric constant close to 230 for Pb(Zr_0.6_Ti_0.4_)O_3_ and 350 for Pb(Zr_0.53_Ti_0.47_)O_3_, reaching different values at room temperature depending on the Zr/Ti ratio and on the dopant. As can be observed, the intrinsic contribution depends only on the crystal structure attaining a higher value at MPB. This is because the existence of thermodynamically equivalent phases in the MPB promotes the polarization rotation, improving dielectric response [7]. The extrinsic response also maximizes at MPB [34], as shown in Figure 1, but is strongly dependent on the dopant. Both the crystal structure and the dopant determine the domain wall contribution. An expected monotonous increasing ε′(*T*) dependence is shown for Nb-doped PZT, while a more complex behavior is displayed for Fe-doped PZT. This different extrinsic response is due to the different character of the defects created by donor and acceptor dopants, as is supported later.

The nonlinear response concept can be experimentally studied by quantifying the instability of the properties when an alternating external stimulus, either electrical or mechanical, is applied to the material. The increment of a complex property *p* = *p*′ − *i*·*p*″, which corresponds either to the permittivity or to the piezoelectric coefficient, refers to its rate of change with increasing amplitude of the driving signal and is denoted as Δ*p* = Δ*p*′ − *i*·Δ*p*″.

**Figure 2:**
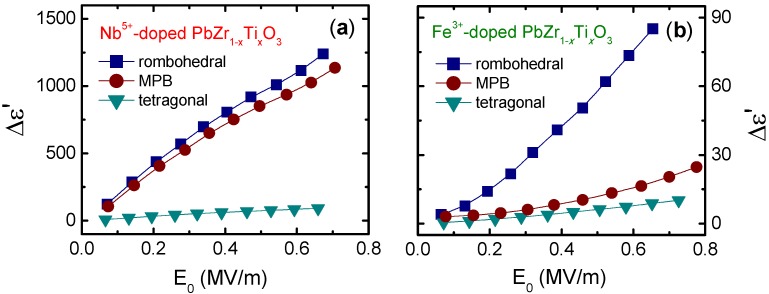
Increments of the dielectric constant as a function of the applied electric field amplitude for Pb(Zr_1-*x*_Ti*_x_*)O_3_ (*x* = 0.40, 0.47, 0.60) doped with (**a**) softener and (**b**) hardener ions. The data were partially extracted from the reference [26] (Copyright AIP Publishing LCC., 2008).

Figure 2 shows the increments of the dielectric constant for Nb- and Fe-doped Pb(Zr_0.60_Ti_0.40_)O_3_ (rhombohedral PZT), Pb(Zr_0.53_Ti_0.47_)O_3_ (MPB PZT) and Pb(Zr_0.40_Ti_0.60_)O_3_ (tetragonal PZT). Here, Δε is calculated from the *D*(*E*) response according to the procedure detailed in [36]. As can be observed, the nonlinear response also depends on the crystal structure and the dopant, and is larger in the rhombohedral composition doped with donor ions. These correlations are discussed below.

### 2.1. Extrinsic Origin of the Nonlinear Response

The extrinsic nature of the nonlinear response was first established over twenty years ago [37]. The temperature-dependent hysteretic response related to nonlinear behavior suggests that the origin of nonlinear contribution is a dynamic phenomenon. The increase in the nonlinear behavior supposes an increase in losses, which are generated from the domain wall motion. Since the nonlinear response is extrinsic in nature, it may be reduced by applying a DC electric field or by decreasing the temperature. Here, the extrinsic origin of the nonlinear response is shown by means of the nonlinear dielectric response measurement in temperature.

**Figure 3:**
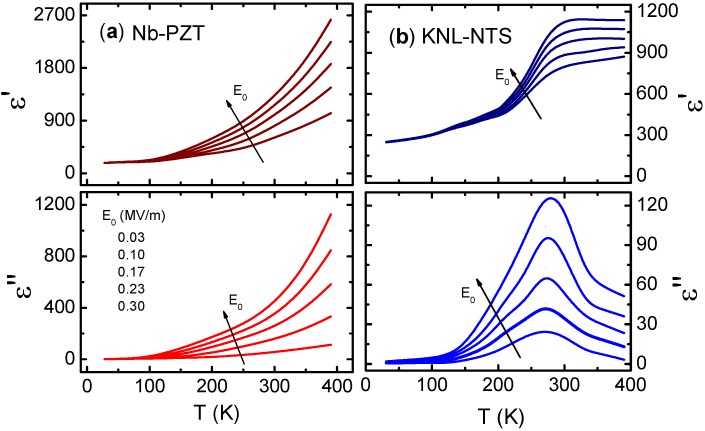
Temperature dependence of real, ε′, and imaginary, ε″, components of the relative dielectric permittivity at 1 kHz as a function of the amplitude of the applied electric field for (**a**) lead-based Nb-doped Pb(Zr_0.60_Ti_0.40_)O_3_ (Nb-PZT) and (**b**) lead-free (K_0.44_Na_0.52_Li_0.04_)(Nb_0.86_Ta_0.10_Sb_0.06_)O_3_ (KNL-NTS). The data were partially extracted from the reference [38] (Copyright AIP Publishing LCC., 2012).

Figure 3 shows the temperature dependence of the relative dielectric permittivity at 1 kHz as a function of the amplitude of the applied electric field for two well-known piezoceramic compositions. At low temperatures (below 150 K), the nonlinear response almost disappears for both materials, which is as expected as a result of the domain wall freezing phenomenon. This means that when only the intrinsic contribution exits, the nonlinear behavior disappears. The nonlinear dielectric response is significant at temperatures at which the domain wall motion contribution becomes important, usually above 150 K. These observations clearly show that the nonlinear behavior is directly related to the extrinsic effect.

### 2.2. Domain Wall Dynamics

The dependence of the extrinsic contribution on the material properties has been well studied by using the Rayleigh model. The Rayleigh law, originally stated in order to describe the magnetic response of a ferromagnetic material to the action of an external magnetic field, has been satisfactorily used to describe the dielectric and piezoelectric nonlinear response in a piezoelectric ceramic [12,26,39]. This model can provide valuable information about the dynamics of domain wall motion. One hypothesis sustaining this model is that the nonlinear behavior is exclusively due to the irreversible motion of domain walls [24]. The model assumes that the response of the material is due to the interaction of the domain walls with the defects of the material. These defects act as randomly distributed pinning centres by hindering the movement of the domain walls. Analysis of the dielectric response in terms of the Rayleigh law enables the contribution of the irreversible movement of domain walls to be studied. Therefore, the reversible domain wall motion contribution leads to deviations from the Rayleigh law predictions [40].

The extrinsic contribution can be evaluated from the increments of the real and imaginary parts of the permittivity as a function of the amplitude of the applied electric field. The Rayleigh model assumes that the increment in the real as well as the imaginary dielectric permittivity linearly depends on the amplitude of the applied electric field, as follows [40]:

(1) $$\text{Δ}{\text{ε}}^{\prime }=\;\text{ε }\times E_{0}$$

(2) $$\text{Δ}{\text{ε}}^{\prime \prime }=\frac{4}{3\text{π}}\times \text{α}\times E_{0}$$

where α is the Rayleigh coefficient and is directly related to the magnitude of the nonlinear response. Moreover, the ratio between the value of imaginary and real increments of dielectric permittivity is a constant that does not depend on the material:

(3) $$m_{\text{ε}}=\frac{\text{Δ}{\text{ε}}^{\prime \prime }}{\text{Δ}{\text{ε}}^{\prime }}=\;\frac{4}{3\text{π}}\;\approx 0.42$$

Fulfilment of the relations Equations (1), (2), and (3) implies a Rayleigh behavior associated to a preponderant irreversible domain wall motion. Rayleigh behavior involves some hypotheses that are not valid for all materials. Results obtained by different authors show that, for example, in hard PZT the dielectric behavior does not linearly depend on the amplitude of the electric field, and that the relation Δε″/Δε′ does not have the value predicted by the Rayleigh model [40,41,42]. In other cases, even when Δε′ and Δε″ are linearly dependent on *E*_0_, a non-Rayleigh behavior associated with a shift away of *m*_ε_ from its theoretical value may occur. Nevertheless, this *m*_ε_ shift provides us with information about the preponderant mechanism in the domain wall dynamic, because *m*_ε_ relates the ratio between reversible and irreversible domain wall motion processes.

**Figure 4:**
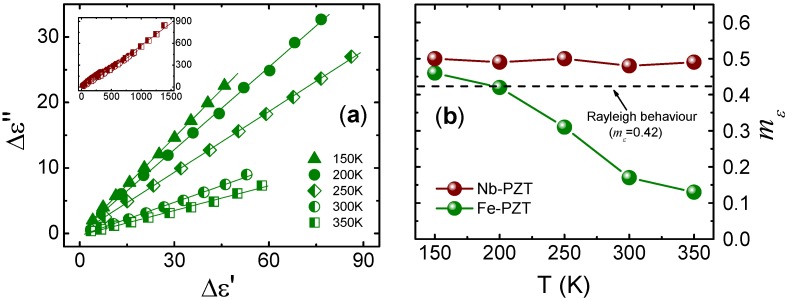
(**a**) Relation between the increments of the imaginary (Δε″) and real (Δε′) parts of the relative permittivity for Fe-doped Pb(Zr_0.60_Ti_0.40_)O_3_ (Fe-PZT) at several temperatures. In the inset, this relation is shown for Nb-doped Pb(Zr_0.60_Ti_0.40_)O_3_ (Nb-PZT) at the same temperatures. Linear fits are shown as solid lines; (**b**) Ratio between the increments of the imaginary and real parts of the permittivity (*m*_ε_) for both Fe-PZT and Nb-PZT at several temperatures. The data were extracted from the reference [38] (Copyright AIP Publishing LCC., 2012).

Figure 4a shows the relation between the increments of the imaginary (Δε′) and real (Δε″) parts of the relative permittivity at different temperatures for Fe-doped (hard) rhombohedral Pb(Zr_0.60_Ti_0.40_)O_3_. This relation for Nb-doped (soft) Pb(Zr_0.60_Ti_0.40_)O_3_ is also shown in the inset. At each temperature, the interval of applied field amplitudes is the same in all cases. As can be observed, there is a linear relation between the real and imaginary parts of the permittivity, which is a common characteristic that has been reported for a wide number of piezoceramics [26,27,29,30,43], even when Δε′ and Δε″ do not depend linearly on the field amplitude. Two features may be highlighted in the Δε″ *versus* Δε′ graph: the distance between two adjacent points of a plot and the slope of the plot. The distance between the points reveals the significance of the nonlinear effect, while the slope of the plot quantifies the *m*_ε_ value. This value indicates that a quantitative relation between the real and imaginary parts of the permittivity exists, *i.e.*, an increment in the dielectric constant implies a given increment of losses.

The *m*_ε_ value as a function of the temperature is shown in Figure 4b for both Fe-doped and Nb-doped Pb(Zr_0.60_Ti_0.40_)O_3_. Note that the *m*_ε_ value remains almost constant for Nb-PZT, while an evolution towards lower values is observed for Fe-PZT. In terms of the Rayleigh model, the *m*_ε_ values for Nb-doped PZT appear to be slightly higher than those expected, thereby revealing the presence of some dissipative processes that have not been taken into account by the model; *i.e.*, processes in which the losses contribute more than that predicted by the Equation (3). Field-induced phenomena, e.g., conductivity effects, may account for the obtained additional dissipation in this material. In any case, and based on the Rayleigh law, the contribution of the irreversible displacement of domain walls appear to be the mechanisms governing the Nb-PZT response for all temperatures. On the other hand, the dielectric response fits the Rayleigh model only for *T* = 200 K in Fe-PZT, thereby showing that the relevant mechanisms governing the dielectric response differ according to the temperature. In this material, there appears to exist a reversible (elastic) domain wall motion mechanism contributing to the dielectric constant, but which has no associated losses [40].

Analysis of the nonlinear dielectric response undoubtedly indicates a possible change in the dynamics of the domain wall motion in Fe-doped PZT. The different temperature dependence of the extrinsic response due to the different domain wall dynamics determines the room temperature properties in PZT piezoceramics [38]. Changes in the domain wall dynamics may be responsible for other singular properties of emerging materials. For example, a change in domain wall dynamics has been detected in the (K,Na)NbO_3_ system when crossing the orthorhombic to tetragonal phase transition [44], which could be decisive for their properties when this phase boundary is located near room temperature.

A wider interpretation of the Rayleigh law was subsequently developed from the Preisach model [45]. The Preisach formalism assumes that a hysteretic system consists of a superposition of independent bi-stable units, each one characterized by two parameters: a bias field, induced in each bi-stable by the action of its environment, and a coercive field, defined as the amplitude of the external field required to switch the bi-stable [46]. From a macroscopic point of view, the system is defined by a distribution function of the parameters allied to each bi-stable.

The Preisach model allows us to describe the Rayleigh empirical relations when the bistable distribution in the two-dimensional space of bias and coercive parameters is taken to be uniform. Then, the Rayleigh law is derived from the Preisach formalism and may be regarded as a particular case of this formalism. The fact that different distribution functions allow us to generate different types of response makes the Preisach model an important tool for describing the nonlinear response of ferroelectric materials [47,48]. However, a restriction exists, and that is that the Preisach formalism is based on a hysteretic system where all contributions to the dielectric or piezoelectric response are directly related to losses. Thus, in this model it is assumed that in any case only irreversible processes contribute to the nonlinear response of the material.

## 3. Nonlinearity in Lead-Based Piezoceramics

### 3.1. Crystallographic Phase Effect

**Figure 5:**
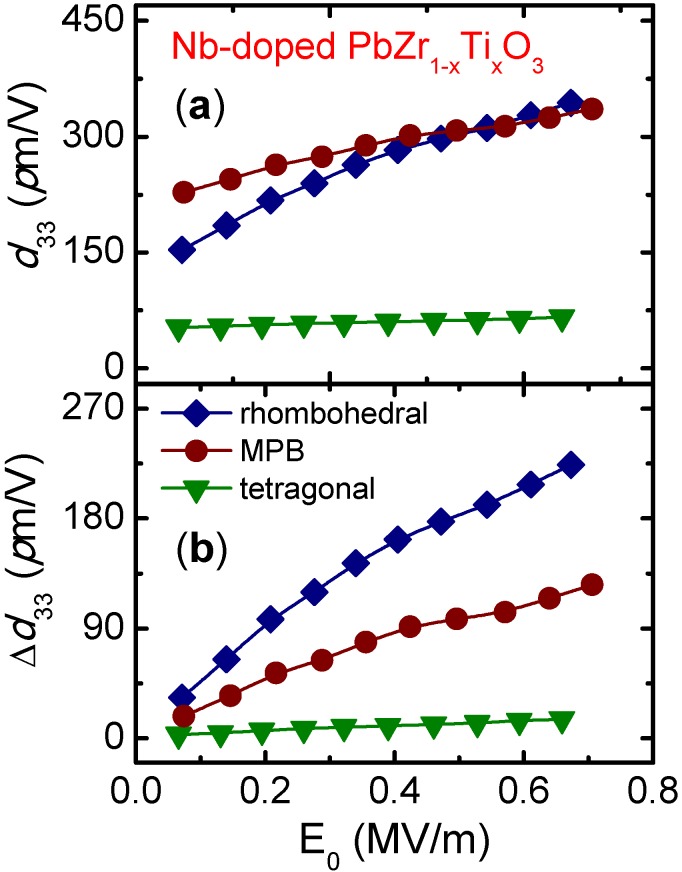
(**a**) Total value and (**b**) increment of the converse piezoelectric coefficient as a function of the applied electric field amplitude for Nb-doped Pb(Zr_1−*x*_Ti*_x_*)O_3_ with *x* = 0.40 (rhombohedral), 0.47 (MPB) and 0.60 (tetragonal). The data were extracted from the reference [26] (Copyright AIP Publishing LCC., 2008).

The formation of the domain configuration in ferroelectrics is mainly determined by the crystal structure. For instance, 90° and 180° domain walls appear in tetragonal materials, while 71°, 109°, and 180° domain walls are allowable in rhombohedral materials. Since the domain wall motion is directly related to the domain configuration, the crystallographic phase has a decisive influence on the extrinsic response and, consequently, on the nonlinear behavior. Figure 5 shows the total value and the increment of the converse piezoelectric coefficient as functions of the amplitude of the applied electric field for donor-doped PZT compositions with different crystallographic phases. As can be observed, the nonlinear response of PZT is strongly dependent on the crystal structure. The increment in dielectric constant and piezoelectric coefficient are greater in rhombohedral materials than in MPB, and in both cases are greater than in tetragonal ones. This is the same observation that can be made from the dielectric response in Figure 2a. Results from the dielectric and piezoelectric response of acceptor-doped PZT with the same nominal compositions show an equivalent tendency [15]. Similar results were also obtained in donor-doped PZT from the direct piezoelectric effect measurements [24]. Analogous observations were reported from the analysis of the dielectric and converse piezoelectric responses of the (1−*x*)BiScO_3_−*x*PbTiO_3_ (BS-PT) ceramic system [49].

Two phenomena may contribute to the dependence of the nonlinear response on the crystallographic phase. Firstly, the amount of spontaneous strain of the crystalline structure may govern the domain wall mobility. The spontaneous strain of the rhombohedral elemental cell is less than 0.5%, while the strain is greater than 2% in the tetragonal cell, reaching 6% for Ti-rich PZT compositions [4]. The high distortion of the tetragonal cell may create strong local internal stress, which will make domain wall motion difficult. On the other hand, the rhombohedral domain walls can move with relative freedom due to the low distortion of the lattice, which scarcely causes internal stress. Secondly, the nonlinear response is enhanced at the rhombohedral phase because more directions are available to be oriented, and therefore the material exhibits a higher extrinsic contribution. A further analysis is required to understand why nonlinear behavior is lower in the MPB than in the rhombohedral phase. A complex domain structure is formed at MPB as a result of the coexistence of tetragonal and rhombohedral domains. The domain coexistence may lead to the appearance of an internal stress that reduces domain wall mobility, as was demonstrated in (K,Na)NbO_3_ based ceramics [50,51]. It is important to point out that the highest value of low-field piezoelectric coefficient is reached at MPB, where the intrinsic contribution plays an important role.

### 3.2. Doping Effects

Compositional engineering by doping is a very active research line for obtaining piezoceramics with enhanced properties. Many papers are published every year on compositional modifications as an approach to improving some specific material property. In particular, the substitution of the A- or B-site of the perovskite structure by lower valence ions generates oxygen vacancies that give rise to the formation of so-called complex defects [52]. These defects operate as pinning centers, thereby making the domain wall movement more difficult [53]. On the other hand, the A- or B-site substitution by higher valence ions generates lead vacancies and reduces oxygen vacancies, thus making domain wall movement easier [52]. So, for instance, in the PZT system the substitution of Zr^4+^ o Ti^4+^ by lower valence ions (e.g., Fe^3+^) hardens the material properties, while higher valence ions (e.g., Nb^5+^) soften the properties.

**Figure 6:**
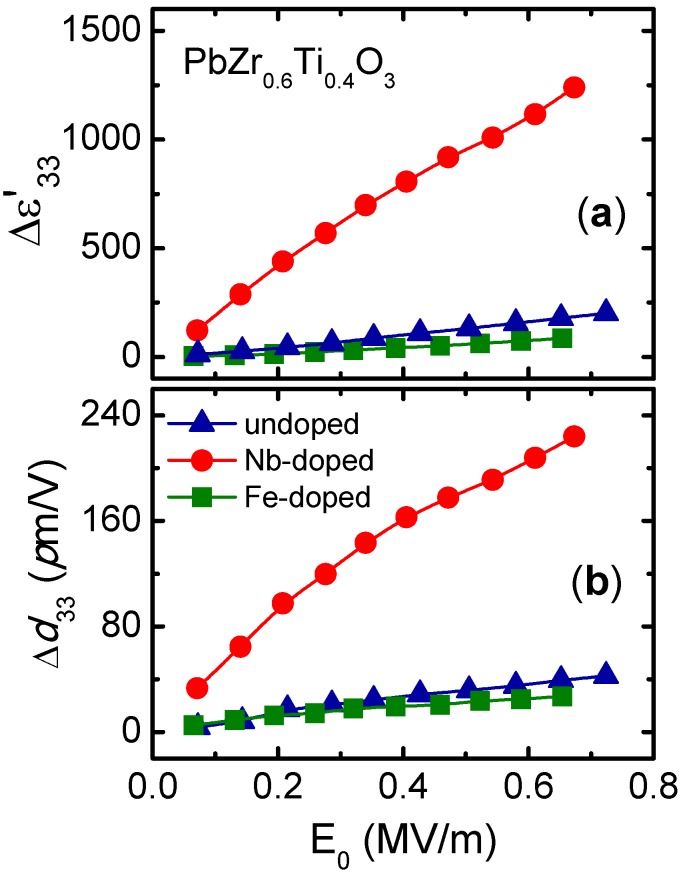
Increments in (**a**) the dielectric constant and (**b**) the converse piezoelectric coefficient as a function of the applied electric field amplitude for undoped, Nb-doped, and Fe-doped Pb(Zr_0.60_Ti_0.40_)O_3_. The data were extracted from the reference [26] (Copyright AIP Publishing LCC., 2008).

Figure 6 shows the role of the hardener and softener substitutions in nonlinear response. As can be observed, as regards the pure material, a significant increase in the dielectric constant and in the piezoelectric coefficient is produced in donor-doped materials, while a decrease in the same is produced in acceptor-doped ones.

It should be noted that the effect of the softener ions is notably greater than that of the hardener ones. These results can be explained by taking into account the interaction of the domain walls with the defects formed by oxygen vacancies. It is well known that during the process of synthesis of PZT, oxygen vacancies are formed as a result of the unavoidable evaporation of lead [54]. These vacancies form complex defects resulting in a hard-type behavior for undoped PZT. The addition of Fe^3+^ ions in substitution for Zr^4+^ or Ti^4+^ ions will increase the oxygen vacancies and lead to the creation of new complex defects. These defects can be oriented in the direction of the applied field and thus make the movement of the domain walls difficult, as pointed out above. On the other hand, the addition of Nb^5+^ ions creates lead vacancies by increasing the mobility of the domain walls as a consequence of the low stress of the lattice. Moreover, the insertion of donor impurities in a perovskite structure will reduce the oxygen vacancies by charge compensation [55], so the pinning of the walls is reduced as a result of the decrease in the concentration of complex defects. Definitively, the decrease of oxygen vacancies seems to be the main reason for the increase in domain wall mobility in donor-doped materials. This hypothesis has been successfully employed in order to reduce the nonlinear response of (K,Na)NbO_3_–based piezoceramics [56]. Furthermore, an adequate softener and hardener co-doping may enable the material properties to be tuned from soft to hard [27].

Recovering Figure 1, different dielectric responses can be detected near room temperature for the same composition, depending on dopant. Anomalous ε′(*T*) is observed in acceptor-doped PZT but not in donor-doped PZT for both rhombohedral and MPB compositions; this was also verified for tetragonal composition [35]. This anomaly, which appears to be intimately related with the acceptor dopant, is a direct manifestation of the domain wall pinning effect.

### 3.3. Grain Size Effect

Piezoelectric devices are not oblivious to the current miniaturization trends in ceramic technology for microelectronics. Microelectromechanical systems, for instance, require ceramic layers with a submicron or even a nanometric grain size [57]. A large number of studies exist that address the grain size effect in BaTiO_3_ (BT), whose properties exhibit a singular grain size dependence that was firstly associated with the evolution of domain structure [58]. A more recent study correlates the grain size with the tetragonal distortion evolution and the ferroelectric properties, revealing an intrinsic size effect [59]. Measurement of the nonlinear piezoelectric response in coarse- and fine-grained BT show that domain wall motion decreases when the grain size is smaller [28]. The published research on grain size effect in BT demonstrates that both intrinsic and extrinsic contributions depend on grain size in perovskite ferroelectrics.

In general, high sensitivity, microstructured piezoceramics undergo a significant functionality reduction when grain size evolves to nanoscale. In soft PZT, for instance, the longitudinal piezoelectric coefficient notably decreases when the grain size moves from micron to submicron size, as a result of the decrease in domain wall mobility [60]. In this context, the scientific challenge is then to obtain high performance piezoceramics with submicron grain sizes close to the nanoscale. Significant advances have been made in this regard in the last decade [61], and a large number of papers have been published related to piezoceramic properties with submicron grain sizes.

The worsening of the functional properties when grain size is decreased to a submicron scale appears mainly to be due to the reduction of domain wall motion as a consequence of the domain wall clamping effect, which implies a decrease in the nonlinear response. The few reported results about the grain size effect on nonlinear response support this hypothesis [24,43]. Therefore, the reduction of grain size decreases the functionality of piezoceramics, but provides more stability to their properties. In any case, further studies are needed in order to effectively research the grain size effect on the nonlinear behavior of piezoelectric ceramics. Furthermore, the nonlinear response analysis could shed new light on the grain size effect on the dynamics of the domain wall motion.

## 4. Nonlinearity in Lead-Free Piezoceramics

The discovery of a (K,Na)NbO_3_–based piezoceramic with a large piezoelectric response [62] triggered a race to replace lead-containing piezoceramics with non-toxic alternatives. This race was also driven by the legislative activity, initially promoted from the European Union, and subsequently followed by other countries, for the purpose of protecting human health as well as the environment by the exclusion or substitution of hazardous substances used in electrical and electronic devices. The intense scientific activity conducted in developing new lead-free piezoceramics has been continuously reviewed in the last decade [63,64,65,66,67,68,69,70,71,72,73,74]. Although the number of the annual refereed publications is extremely high (about 400 refereed papers [74]), only few reports can be found related to field-induced instability properties in lead-free compositions. Here, some aspects related to nonlinear behavior of representative lead-free piezoceramics are discussed.

### 4.1. KNN-Based Compositions

(K,Na)NbO_3_-based systems have attracted an indisputable interest as the most promising candidates for commercially viable lead-free piezoceramics [73,74]. Among these, the (K,Na)NbO_3_–LiTaO_3_–LiSbO_3_, and in particular the (K_0.44_Na_0.52_Li_0.04_)(Nb_0.86_Ta_0.10_Sb_0.04_)O_3_ (KNL-NTS) composition [75] is probably the most workable lead-free piezoelectric system known to date. The origin of the high piezoelectric activity in this compound seems to reside in a mixture of a compositional-driven (morphotropic) and temperature-driven (polymorphic) phase transition regions [76]. Unfortunately, its properties are not suitable for all end uses. For instance, KNL-NTS exhibits a relatively high piezoelectric coefficient and coupling factor at room temperature, but also high losses and a low quality factor. Furthermore, the properties are notably temperature- and field-dependent.

**Figure 7:**
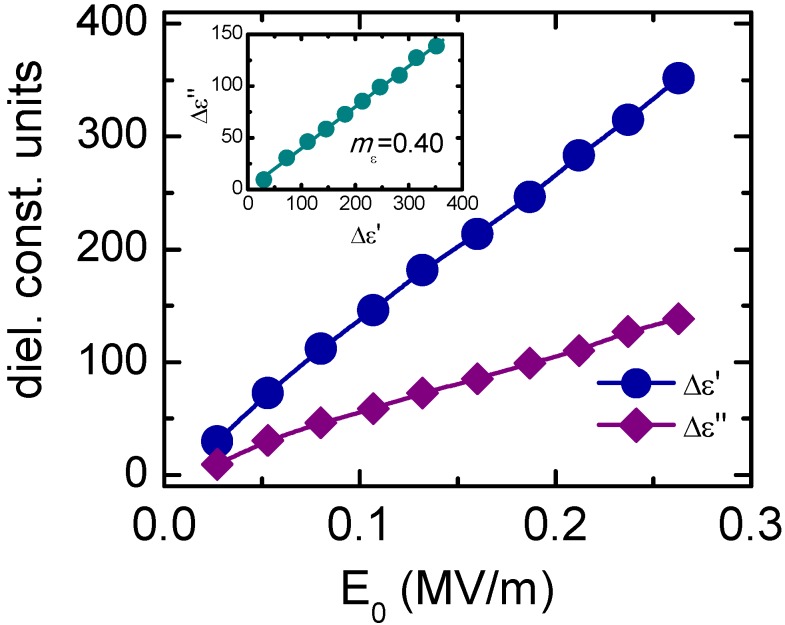
Increments of the real (Δε′) and imaginary (Δε″) parts of the relative permittivity for KNL-NTS at room temperature. The inset shows the relation between Δε″ and Δε′, and the ratio *m*_ε_ = Δε″/ Δε′ is reported. Figure reprinted from reference [30] (Copyright IOP Publishing Ltd., 2009).

Figure 7 shows the relative increases of the real (Δε′) and imaginary (Δε″) parts of the relative permittivity as a function of the electric field amplitude for a KNL-NTS piezoceramic obtained by a conventional solid state synthesis process [77]. Both Δε′ and Δε″ show a linear dependence on the electric field amplitude, which corresponds to the Rayleigh-type behavior (Equations (1) and (2)). In addition, the ratio *m*_ε_ = 0.40 also verified a Rayleigh-type nonlinear response (Equation (3)) for KNL-NTS. Thus, the irreversible domain wall motion is a mechanism that governs nonlinear response in KNL-NTS ceramics at room temperature, which is similar to that in soft PZT ceramics.

Additional efforts are needed in order to reduce nonlinear behavior in KNN-based materials [56,78]. From this perspective, good results are expected by means of hardener substitutions, such as occur in PZT, although some structural and electrical aspects remain controversial as regards the role of dopants in the KNN system. Significant advances have been reported through Cu-doping. It has been shown that Cu-doped KNN-modified compounds may exhibit typical characteristics of hard behaviour [79,80,81,82]. Nevertheless, few reports exist about the stability of the properties of these materials.

### 4.2. Other Lead-Free Piezoceramics

In the race to replace the PZT, (Bi_0.5_Na_0.5_)TiO_3_-BaTiO_3_ (BNT-BT) and (Ba,Ca)(Zr,Ti)O_3_ (BCZT) based materials have also attracted a great deal of interest. The (1−*x*)BNT-*x*BT system sparked interest because a MPB was verified for *x* = 0.06–0.07 [83]. However, the properties of this MPB system are restricted due to their temperature dependence. In particular, a low depolarization temperature is shown as a manifestation of a ferroelectric to antiferroelectric phase transition [83]. Once they have formed ternary systems, BNT-BT-based materials may become candidates for low temperature actuator applications, as in the case of BNT-BT-KNN [74].

**Figure 8:**
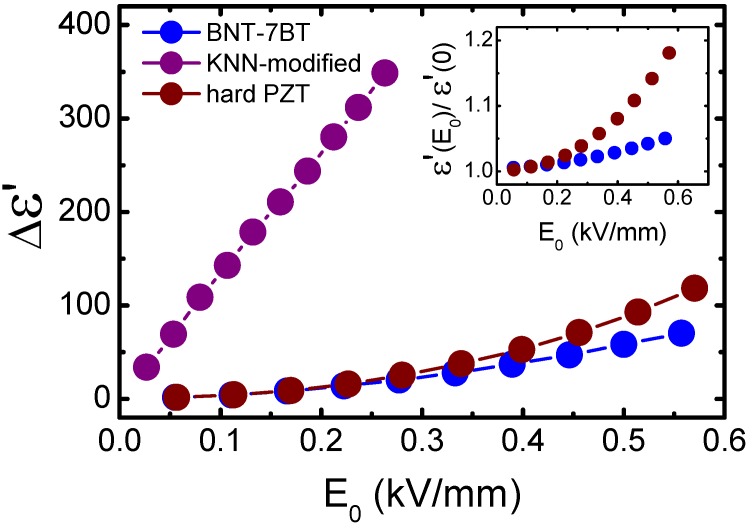
Increments of the dielectric constant as a function of the applied electric field amplitude for (K_0.44_Na_0.52_Li_0.04_)(Nb_0.86_Ta_0.10_Sb_0.04_)O_3_ (KNN-modified), 0.93(Bi_0.5_Na_0.5_)TiO_3_–0.07BaTiO_3_ (BNT-7BT) and Pz26 (hard commercial PZT, from Ferroperm). The inset shows the relative increment of the dielectric constant for BNT-7BT and hard PZT.

A comparative analysis of the dielectric constant stability of three representative piezoceramics is displayed in Figure 8. The increment of the dielectric constant as a function of the applied electric field for 0.93(Bi_0.5_Na_0.5_)TiO_3_–0.07BaTiO_3_ (BNT-7BT) compound is plotted together with the values for KNL-NTS (KNN-modified) and a commercial hard PZT (Pz26, from Ferropern). BNT-7BT is a room temperature MPB BNT-BT obtained by conventional solid state reaction, which exhibits longitudinal piezoelectric coefficient *d*_33_ around 180 pC/N. A high stability of the dielectric constant is observed in this compound, which is even higher than that found in a commercial hard PZT. The inset data verifies the same insight from the relative increment of the dielectric constant. Results from the piezoelectric response (not shown here and yet to be unpublished) confirm the high stability of this system. Selected compositions, which show moderate but not low piezoelectric coefficient, could be candidates for specific room temperature applications since their properties are stable. In any event, further studies are required in order to elucidate the microscopic origin of the high stability observed in BNT-BT.

On the other hand, greater research activity is expected to be conducted on the (Ba,Ca)(Zr,Ti)O_3_ lead-free system in the coming years. Exceptional piezoelectric properties were reported in (1−*x*)Ba(Zr_0.2_Ti_0.8_)O_3_−*x*(Ba_0.7_Ca_0.3_)TiO_3_ [84] that opened a new research era on lead-free piezoelectrics. The BCZT system also shows a low Curie temperature, but deserves a lot of attention for room temperature usage [74]. Nowadays, much research in this system is being carried out. However, the first comprehensive studies of field-induced instability of properties are still to come.

## 5. Final Remarks and Outlook

Nonlinear response analysis is a powerful tool for studying the domain wall dynamics in ferroelectric materials as well as for elucidating the role that extrinsic behavior plays in the material response. This type of characterization may be necessary to evaluate the performance of emerging compositions, in particular, the new lead-free systems. Not only are high values of electromechanical coefficients desirable, but also a high stability of these properties is needed to effectively replace lead-based compositions in high power applications.

The extrinsic nature of the nonlinear response implies that the domain wall motion not only improves piezoelectric properties, but also maximizes the field-induced instability of these properties. The crystal structure determines domains configuration, and consequently the domain wall motion and dynamics. Compositional engineering by doping is an effective method to improve particular properties for specific applications, because this is a real way to modify domain wall dynamics in perovskite ferroelectrics. Further studies related to the grain size effect on the nonlinear response of piezoceramics are required from both the fundamental and applied point of view. From the fundamental point of view, nonlinear response analysis may enable the extrinsic character of the size effect to be assessed, and thus correlate it with micro(nano)structural aspects such as the formation of complex domain structures into submicron grains.
